# Socio-demographic and economic inequalities in modern contraception in 11 low- and middle-income countries: an analysis of the PMA2020 surveys

**DOI:** 10.1186/s12978-020-00931-w

**Published:** 2020-06-01

**Authors:** Cauane Blumenberg, Franciele Hellwig, Fernanda Ewerling, Aluísio J. D. Barros

**Affiliations:** 1 International Center for Equity in Health (ICEH), Federal University of Pelotas, Rua Marechal Deodoro 1160, 3o piso, Pelotas, RS 96020-220 Brazil; 2 Postgraduate Program in Epidemiology, Federal University of Pelotas, Pelotas, RS Brazil

**Keywords:** Family planning, Contraception, Health equity

## Abstract

**Background:**

Contraception is a key component of sustainable development, empowering women, reducing the risk of maternal and child mortality and promoting economic growth. It is part of the Sustainable Development Goals agenda, where the aim is to achieve universal access to sexual and reproductive health. Our objective was to evaluate trends and inequalities in modern contraceptive prevalence, and according to the type of modern contraceptive, in 11 low- and middle-income countries that are partners of the Family Planning 2020 initiative.

**Methods:**

Analyses were performed using 62 Performance Monitoring and Accountability 2020 (PMA2020) surveys from 11 countries. Forty surveys were nationally representative, while 22 had regional coverage. Regional surveys were analyzed separately, totalizing 15 geographies from 11 countries. We described trends on modern contraceptive prevalence, and its subtypes (short- and long-acting reversible contraceptives, and permanent methods), by calculating absolute average annual changes. Absolute inequalities on the prevalence of modern contraceptives were assessed for the most recent survey of each geography using the slope index of inequality, and according to wealth, education and age.

**Results:**

The overall prevalence of modern contraception increased in most geographies analyzed, reaching a 7.2 percentage points increase per year in Lagos, Nigeria. This increase was mostly influenced by the long-acting reversible contraceptives, which increased in 73% of the geographies. Although the largest share of modern contraception is represented by short-acting reversible contraceptives, these are reducing and giving space for the long-acting methods. The exception was Rajasthan, India, where the permanent methods accounted for 70% of the modern contraception share, and their prevalence was almost 40%. Inequalities were identified in favor of richer, older and better educated women.

**Conclusions:**

Out of the 15 geographies analyzed, 11 demonstrated an increase in overall modern contraceptive use – mainly driven by the uptake of long-acting reversible contraception. However, even in the groups with the highest prevalence, modern contraceptive use was at most 60% in most geographies. So, we are far from reaching the desired universal coverage proposed by the Sustainable Development Goals.

## Plain english summary

Contraception is a key aspect of sustainable development, helping people to achieve reproductive wishes and contributing to reduce abortions and the risk of maternal and child mortality. Considering these benefits, the United Nations aim to achieve universal access to sexual and reproductive health by 2030 as part of the Sustainable Development Goals agenda. The number of women in need of contraception that are using contraceptives is increasing worldwide, but these numbers may vary depending on the region of the country, and on the sociodemographic characteristics of women. Our objective was to assess how modern contraceptive use changed from 2013 to 2018 in 11 low- and middle-income countries, and to analyze differences on modern contraceptive use according to the women’s sociodemographic characteristics. We divided modern contraceptives into three subtypes: short-acting reversible contraceptives (e.g. pills, condoms, etc.), long-acting reversible contraceptives (e.g. subdermal implants and intrauterine devices) and permanent methods (e.g. sterilization). Our results showed that, in general, the use of modern contraceptives increased. This increase was mostly influenced by a reduction on the use of short-acting contraceptives, which are giving space for the long-acting contraceptives. In Rajasthan, India, the scenario was different. There, sterilizations were the most commonly used contraceptive method. Regardless of the subtype of modern contraceptive, the proportions of richer, older and better educated women using modern contraceptives were higher compared to poorer, younger and worst educated women, respectively. There was an important progress on the use of modern contraceptives in the countries analyzed, but we are still far from reaching the universal coverage.

## Background

The importance of universal access to sexual and reproductive health is already well recognized as a crucial component of sustainable development [1]. By allowing people to achieve their wishes regarding the number and spacing of children, contraception can reduce abortions, reduce the risk of maternal and child mortality, and promote economic growth and women’s empowerment [2, 3].

Despite the increase on contraceptive prevalence and demand for family planning satisfied worldwide, over 200 million women who want to avoid pregnancy in developing countries are still not using modern contraceptive methods [2, 4]. While the coverage of demand for family planning satisfied with modern methods is around 70% in South Asia region, it is as low as 33% in West & Central Africa – the poorest region in the world, with the highest fertility rates, and lower levels of women empowerment [5, 6]. Within-country inequalities are also observed, with lower levels of coverage among poorer, uneducated, rural and younger women [5, 7].

In order to reduce inequalities, empower women and increase family planning coverage, the United Nations’ Sustainable Development Goals 3 and 5 aim to achieve universal access to sexual and reproductive health [8, 9]. To support reaching these goals, a partnership called Family Planning 2020 (FP2020) was built by federal governments, civil society, private companies and foundations. These actors made commitments to address the delivery, financing and policy to enable that 120 million additional women and girls in 69 of the world’s poorest countries have access to modern contraception by 2020 [10]. Since its launch, several countries made commitments and launched actions to remove socio-cultural barriers and to improve access to sexual and reproductive health services [11]. Recent evidence shows that priority FP2020 countries, chosen due to their large population, are making satisfactory progress toward achieving the goal. Among these countries are Burkina Faso, Ghana and Nigeria [10, 12]. Although positive at national level, there is no evidence on how progress occurred in subgroups of women, notably those that are usually harder to reach, such as women living in rural areas and with low level of education. Thus, it is important to evaluate how the modern contraceptive prevalence varies within these countries, according to the socio-demographic and economic characteristics of the population.

Our objective was to evaluate the trends of modern contraceptive prevalence (mCPR) in 11 FP2020 priority countries and the related socio-demographic and economic inequalities using data from the Performance Monitoring and Accountability 2020 (PMA2020) surveys. The choice of PMA2020 surveys was because they are carried out much more frequently than other families of household surveys and can offer more detailed perception of trends over time. Given the great variability in the mix of contraceptives used in each country, we also analyzed trends and inequalities according to the subtype of modern contraceptive being used (short-acting reversible methods (SARC), long-acting reversible methods (LARC), and permanent methods (PERM)).

## Methods

The PMA2020 is a series of surveys directed and supported by the Bill & Melinda Gates Foundation at the Johns Hopkins Bloomberg School of Public Health, and Jhpiego, a non-profit health organization affiliated with Johns Hopkins. Also, national partners collaborate in each country, being responsible for the data collection [13]. It was created to monitor the progress of 11 low- and middle-income countries (LMICs) in achieving the FP2020 goal. The surveys were designed to provide up to date information about contraceptive prevalence every 6 months to 1 year for each selected geography. Nine of these countries are from Africa (Burkina Faso, Congo DR, Côte d’Ivoire, Ethiopia, Ghana, Kenya, Niger, Nigeria and Uganda) and two from Asia (India and Indonesia). These countries are covered by PMA2020 surveys, because they are among the most populous LMICs that have committed to the FP2020 initiative. Data collection is done by trained women residing near or within selected enumeration areas (clusters) of each country, using smartphones. Prior to data collection, these women are responsible for listing all households located within an enumeration area. Then, 33 to 44 households from each of these enumeration areas are randomly selected to be part of the PMA2020 sample. All women of reproductive age (15 to 49 years) residing in the selected households are eligible to be interviewed. The interview consists of two main questionnaires: (i) the household questionnaire, collecting information about the household characteristics and basic information about the characteristics of all individuals living in the household; and (ii) the women’s questionnaire, which collects detailed information about health and family planning. PMA2020 data is public available upon request [13].

We analyzed all 62 surveys that were available until April 2020 from the 11 countries monitored by the PMA2020, covering the period between 2013 and 2018 (see details in Table 1). The number of available surveys differed for each country. Out of the 62 surveys, 40 were nationally representative (64.5%) and 22 had regional coverage (35.5%). In Congo DR, seven surveys covered the Kinshasa region and four surveys covered the Kongo Central region. In India, the four surveys carried out covered the Rajasthan state. Two surveys from Niger had national coverage, and another three covered the capital district Niamey exclusively. Finally, three surveys from Nigeria had national coverage, two covered the Kaduna state and the remaining two were restricted to the Lagos state. In our study, we analyzed regional surveys separately, considering them as different surveys compared to national surveys conducted in the same country. For this reason, we report on 15 geographies from 11 countries.

**Table 1:** List Of PMA2020 surveys analyzed

Country (Region)	Sample coverage	Starting years
Burkina Faso	National	2014, 2015, 2016, 2016, 2017, 2018
Congo DR (Kinshasa)	Regional	2013, 2014, 2015, 2015, 2016, 2017, 2018
Congo DR (Kongo Central)	Regional	2015, 2016, 2017, 2018
Côte d’Ivoire	National	2017, 2018
Ethiopia	National	2014, 2014, 2015, 2016, 2017, 2018
Ghana	National	2013, 2014, 2014, 2015, 2016, 2017
India (Rajasthan)	Regional	2016, 2017, 2017, 2018
Indonesia	National	2015, 2016
Kenya	National	2014, 2014, 2015, 2015, 2016, 2017, 2018
Niger	National	2016, 2017
Niger (Niamey)	Regional	2015, 2016, 2018
Nigeria	National	2016, 2017, 2018
Nigeria (Kaduna)	Regional	2014, 2015
Nigeria (Lagos)	Regional	2014, 2015
Uganda	National	2014, 2015, 2015, 2016, 2017, 2018

The main outcome analyzed in our study was the prevalence of modern contraceptive, estimated by dividing the number of women that reported using (or whose partner was using) a modern contraceptive method by the total number of women in reproductive age (15–49 years) that were sexually active. Women were considered sexually active either if they were married or living with partner or if they reported having had sex in the last month. Modern contraceptive methods were classified according to Hubacher and Trussel’s proposed definition: “a product or medical procedure that interferes with reproduction from acts of sexual intercourse” [14]. This includes short-acting reversible contraceptives (oral contraceptive pills, female and male condoms, injectables, emergency contraception, diaphragms, and spermicidal agents), long-acting reversible contraceptives (intrauterine devices and subdermal implants) and permanent methods (female and male sterilization). This definition does not consider lactational amenorrhea and standard days as modern contraceptive methods. Our preference for this definition lies on the fact that many women in LMICs will have difficulty ensuring periods of abstinence, in understanding the different levels of risk of pregnancy along the cycle and identifying an atypical period after pregnancy.

Inequalities in the prevalence of modern contraceptive were assessed according to women’s age (15–17; 18–19; 20–34; 35–49 years), educational level (none; primary; secondary or higher), and wealth quintiles. For sake of simplicity and to facilitate presentation of results, inequalities were analyzed only considering the most recent survey of each geography. Wealth was based on a continuous score provided with the PMA2020 surveys, which was then divided into quintiles, where Q1 represents the poorest 20% households and Q5 the wealthiest 20%. This score is calculated considering the ownership of a comprehensive list of assets, and characteristics of the household, such as wall, floor and roof material, sanitation and water source.

For the trend analyses, some countries had two surveys in the same year, thus we present results with the year and the median month between the starting and ending month of the survey in that year. We used variance weighted least squares regression to estimate the absolute average annual change (AAAC) (in percentage points) of the prevalence of modern contraceptives and its subtypes (SARC, LARC and PERM). In this model the outcome is the prevalence of modern contraceptive in each survey, the predictor is the time (year/month) and the estimates are weighted by the standard error of the prevalence estimated for each time point. Besides taking into account the variability of the estimated prevalence, the variance weighted least squares also allowed us to account for the varying time gaps between consecutive surveys in each country. Similar models were used to estimate the AAAC of the share of each mCPR subtype. The share was calculated as the proportion of women using each subtype of modern contraceptive among those using modern contraception.

Absolute inequalities of the prevalence of mCPR, and its subtypes, were calculated using the slope index of inequality (SII). The SII was calculated through a logistic regression model. For wealth quintiles, the SII estimates the absolute difference in the prevalence of interest between the top and bottom of the wealth distribution, measured in percentage points [15]. The SII was also estimated according to educational level (indicating the absolute difference in the prevalence of interest between women that have secondary or higher education and women with no education) and age (depicting the absolute difference in the prevalence of interest between women aged 15–17 and 35–49). More details about the SII can be obtained elsewhere [15].

Estimates for groups with less than 25 women are not presented in the results and were not considered in the analyses due to their potential low precision. All analyses took into account the study sample designs (sampling weights, clustering and stratification) and were conducted using Stata 15.1 [16].

## Results

Our analyses covered 11 countries but are divided into 15 geographies given some surveys had subnational coverage. We analyzed a total of 190,813 women of reproductive age (15–49 years) that were sexually active. The overall prevalence of modern contraceptive methods increased throughout the years in most geographies (Table 2). The increase, measured by the AAAC, ranged from 0.9 percentage points per year in Ethiopia to 7.2 percentage points per year in Lagos, Nigeria. Niger (Niamey; AAAC = 1.4; 95% CI: − 0.2; 3.0), Congo DR (Kongo Central; AAAC = 0.0; 95% CI: − 1.1; 1.1), Côte d’Ivoire (AAAC = − 0.9; 95% CI: − 3.8; 2.1) and Indonesia (AAAC = − 0.6; 95% CI: − 2.3; 1.1) did not present any change, since their confidence intervals are compatible with a stability scenario. None of the geographies presented evidence of a reduction in modern contraceptive prevalence.

**Table 2:** Prevalence and absolute average annual change (AAAC) of modern contraceptive (mCPR) use and its subtypes by geography using PMA2020 surveys

Country	Year/month	Modern contraceptive prevalence		Modern contraceptive subtype prevalence		
		mCPR (95 CI)	AAAC (95 CI)	SARC (95 CI)	LARC (95 CI)	PERM (95 CI)
**Burkina Faso**						
National	2014/11	18.4 (16.3; 20.7)	3.7 (3.0; 4.3)	9.8 (8.3; 11.6)	8.5 (7.0; 10.2)	0.2 (0.0; 0.7)
National	2015/05	21.0 (18.8; 23.3)		12.3 (10.7; 14.2)	8.8 (7.4; 10.5)	0.1 (0.0; 0.3)
National	2016/03	25.1 (23.2; 27.1)		13.8 (12.4; 15.4)	12.0 (10.7; 13.6)	0.0 (0.0; 0.1)
National	2016/12	25.3 (23.4; 27.3)		13.4 (12.0; 14.9)	12.3 (10.9; 13.8)	0.0 (0.0; 0.1)
National	2017/11	30.9 (28.9; 32.9)		14.8 (13.3; 16.4)	16.2 (14.7; 17.9)	0.1 (0.0; 0.3)
National	2018/12	31.9 (29.9; 34.0)		17.2 (15.6; 18.8)	14.7 (13.2; 16.3)	0.1 (0.0; 0.3)
**Congo DR**						
Kinshasa	2013/11	20.3 (18.0; 22.8)	2.6 (2.0; 3.3)	18.2 (16.0; 20.7)	1.6 (1.0; 2.6)	0.4 (0.2; 1.0)
Kinshasa	2014/09	20.2 (18.4; 22.1)		15.0 (13.5; 16.8)	5.1 (4.2; 6.2)	0.3 (0.1; 0.7)
Kinshasa	2015/05	22.7 (20.5; 25.0)		17.8 (15.8; 20.0)	4.4 (3.5; 5.7)	0.5 (0.2; 1.1)
Kinshasa	2015/11	27.1 (25.0; 29.2)		20.5 (18.6; 22.5)	6.1 (5.1; 7.4)	0.6 (0.4; 1.2)
Kinshasa	2016/09	27.0 (24.5; 29.7)		20.0 (17.8; 22.5)	6.7 (5.3; 8.3)	0.7 (0.3; 1.6)
Kinshasa	2017/09	28.6 (25.7; 31.7)		19.0 (16.6; 21.7)	9.5 (7.6; 11.8)	0.5 (0.1; 1.6)
Kinshasa	2018/11	32.8 (30.0; 35.8)		22.5 (20.1; 25.2)	10.1 (8.3; 12.3)	0.5 (0.2; 1.1)
Kongo Central	2015/12	21.4 (19.2; 23.9)	0.0 (−1.1; 1.1)	18.4 (16.3; 20.7)	1.3 (0.8; 2.1)	2.1 (1.4; 3.0)
Kongo Central	2016/08	19.3 (17.0; 21.9)		16.3 (14.1; 18.7)	1.8 (1.2; 2.6)	1.3 (0.7; 2.3)
Kongo Central	2017/09	18.6 (16.3; 21.3)		15.4 (13.3; 17.9)	2.7 (1.8; 3.9)	0.7 (0.3; 1.5)
Kongo Central	2018/11	22.0 (19.5; 24.8)		17.3 (15.0; 19.9)	4.7 (3.5; 6.2)	0.7 (0.3; 1.5)
**Côte d’Ivoire**						
National	2017/09	23.7 (21.6; 25.9)	−0.9 (−3.8; 2.1)	20.9 (19.0; 23.0)	3.0 (2.2; 4.1)	0.2 (0.1; 0.6)
National	2018/07	22.8 (20.9; 24.9)		19.7 (17.9; 21.7)	3.3 (2.6; 4.2)	0.0 (0.0; 0.0)
**Ethiopia**						
National	2014/02	32.7 (30.7; 34.8)	0.9 (0.3; 1.4)	26.9 (25.1; 28.9)	5.8 (5.0; 6.9)	0.1 (0.1; 0.2)
National	2014/11	34.0 (31.9; 36.1)		26.0 (24.2; 28.0)	7.8 (6.7; 9.1)	0.3 (0.1; 0.8)
National	2015/04	36.3 (34.6; 38.0)		27.7 (26.1; 29.3)	8.5 (7.6; 9.5)	0.5 (0.3; 0.8)
National	2016/03	37.7 (35.9; 39.4)		28.4 (26.8; 30.0)	9.8 (8.8; 10.9)	0.3 (0.2; 0.6)
National	2017/05	35.3 (33.6; 37.1)		25.6 (24.0; 27.1)	9.7 (8.7; 10.8)	0.3 (0.1; 0.6)
National	2018/06	37.7 (36.0; 39.5)		27.0 (25.4; 28.6)	10.4 (9.4; 11.5)	0.5 (0.3; 0.9)
**Ghana**						
National	2013/09	17.8 (16.3; 19.5)	2.6 (2.1; 3.1)	14.1 (12.8; 15.6)	3.3 (2.7; 4.2)	0.4 (0.2; 0.8)
National	2014/03	17.4 (15.9; 19.0)		13.8 (12.5; 15.3)	3.4 (2.7; 4.2)	0.4 (0.2; 0.8)
National	2014/11	20.2 (18.5; 22.1)		15.6 (14.1; 17.3)	4.1 (3.3; 5.2)	0.6 (0.4; 1.0)
National	2015/05	27.9 (26.1; 29.9)		21.9 (20.2; 23.7)	5.9 (5.0; 7.0)	0.9 (0.6; 1.4)
National	2016/09	26.3 (24.3; 28.4)		19.2 (17.4; 21.1)	6.6 (5.6; 7.7)	0.9 (0.5; 1.4)
National	2017/09	26.1 (24.4; 27.9)		16.9 (15.4; 18.4)	8.5 (7.4; 9.6)	1.3 (0.9; 1.8)
**India**						
Rajasthan	2016/07	50.7 (49.0; 52.4)	3.6 (2.4; 4.8)	19.6 (18.3; 21.0)	2.0 (1.6; 2.5)	30.8 (29.2; 32.4)
Rajasthan	2017/03	52.7 (51.1; 54.3)		15.3 (14.2; 16.6)	1.4 (1.0; 1.8)	36.4 (34.9; 38.0)
Rajasthan	2017/08	55.7 (54.1; 57.3)		17.9 (16.7; 19.2)	1.7 (1.3; 2.1)	36.9 (35.3; 38.4)
Rajasthan	2018/05	57.9 (56.2; 59.5)		17.5 (16.2; 18.8)	1.9 (1.5; 2.5)	39.1 (37.5; 40.8)
**Indonesia**						
National	2015/06	59.1 (57.9; 60.4)	−0.6 (−2.3; 1.1)	46.8 (45.5; 48.1)	9.1 (8.4; 9.9)	3.7 (3.3; 4.2)
National	2016/11	58.5 (57.3; 59.8)		45.4 (44.1; 46.6)	9.5 (8.8; 10.3)	3.9 (3.4; 4.4)
**Kenya**						
National	2014/06	51.8 (49.4; 54.2)	1.2 (0.6; 1.7)	38.3 (36.0; 40.7)	12.2 (10.7; 13.9)	1.9 (1.4; 2.5)
National	2014/11	53.0 (50.7; 55.2)		38.4 (36.2; 40.6)	12.6 (11.2; 14.2)	3.2 (2.5; 4.1)
National	2015/06	57.4 (55.2; 59.5)		40.4 (38.2; 42.5)	16.1 (14.5; 17.9)	2.6 (2.0; 3.3)
National	2015/11	60.9 (58.8; 63.0)		40.3 (38.2; 42.4)	19.6 (17.8; 21.5)	2.8 (2.2; 3.4)
National	2016/11	58.4 (56.7; 60.0)		37.3 (35.7; 38.9)	20.2 (18.9; 21.7)	2.7 (2.2; 3.4)
National	2017/11	57.7 (56.0; 59.3)		34.6 (33.0; 36.2)	21.3 (19.9; 22.7)	2.4 (1.9; 3.0)
National	2018/11	59.2 (57.4; 60.9)		32.8 (31.2; 34.5)	24.5 (23.0; 26.1)	2.4 (1.9; 3.1)
**Niger**						
National	2016/04	14.7 (12.8; 16.9)	3.1 (0.3; 6.0)	12.4 (10.6; 14.4)	2.1 (1.4; 3.2)	0.2 (0.1; 0.7)
National	2017/07	18.0 (16.0; 20.1)		14.7 (12.9; 16.7)	3.3 (2.5; 4.3)	0.1 (0.0; 0.4)
Niamey	2015/07	28.3 (25.3; 31.5)	1.4 (−0.2; 3.0)	22.8 (20.0; 25.7)	5.8 (4.4; 7.6)	0.4 (0.1; 1.3)
Niamey	2016/11	31.1 (28.1; 34.3)		23.1 (20.4; 26.1)	10.1 (8.2; 12.3)	1.3 (0.8; 2.3)
Niamey	2018/08	32.5 (29.0; 36.3)		21.6 (18.7; 24.8)	11.3 (8.9; 14.4)	0.1 (0.0; 0.8)
**Nigeria**						
National	2016/05	17.6 (16.5; 18.7)	1.6 (0.8; 2.4)	14.4 (13.4; 15.4)	3.3 (2.8; 3.9)	0.3 (0.2; 0.5)
National	2017/04	17.6 (16.5; 18.7)		13.7 (12.7; 14.7)	3.8 (3.3; 4.3)	0.5 (0.3; 0.8)
National	2018/04	20.9 (19.7; 22.1)		15.6 (14.6; 16.6)	5.2 (4.6; 5.9)	0.5 (0.3; 0.8)
Kaduna	2014/09	10.0 (8.7; 11.4)	5.4 (2.9; 7.9)	7.8 (6.7; 9.0)	2.1 (1.6; 2.9)	0.1 (0.0; 0.5)
Kaduna	2015/08	15.3 (13.3; 17.6)		11.6 (9.9; 13.6)	4.5 (3.3; 6.2)	0.7 (0.3; 1.7)
Lagos	2014/01	18.6 (15.4; 22.2)	7.2 (2.7; 11.7)	17.1 (14.1; 20.6)	1.5 (0.7; 3.1)	0.0
Lagos	2015/09	25.7 (22.9; 28.7)		20.5 (17.9; 23.3)	4.4 (3.3; 6.0)	0.9 (0.4; 1.9)
**Uganda**						
National	2014/05	25.4 (23.6; 27.3)	2.4 (1.8; 3.1)	19.9 (18.3; 21.7)	4.1 (3.3; 5.0)	1.8 (1.3; 2.5)
National	2015/01	31.5 (29.6; 33.6)		23.6 (21.7; 25.5)	5.8 (4.9; 6.9)	2.5 (1.8; 3.3)
National	2015/09	30.9 (28.9; 32.8)		24.0 (22.2; 25.8)	5.3 (4.5; 6.3)	1.9 (1.4; 2.5)
National	2016/04	32.2 (30.3; 34.1)		23.9 (22.3; 25.7)	5.7 (4.8; 6.6)	2.8 (2.2; 3.6)
National	2017/04	34.6 (32.5; 36.7)		24.6 (22.8; 26.6)	7.5 (6.5; 8.8)	2.5 (1.9; 3.3)
National	2018/04	36.2 (34.1; 38.3)		23.3 (21.5; 25.2)	10.3 (9.1; 11.6)	2.8 (2.1; 3.6)

*AAAC* absolute average annual change in percentage points, *CI*: confidence interval, *LARC* long-acting reversible contraceptive, *mCPR* modern contraceptive, *PERM* permanent method, *SARC* short-acting reversible contraceptive

**Fig. 1:**
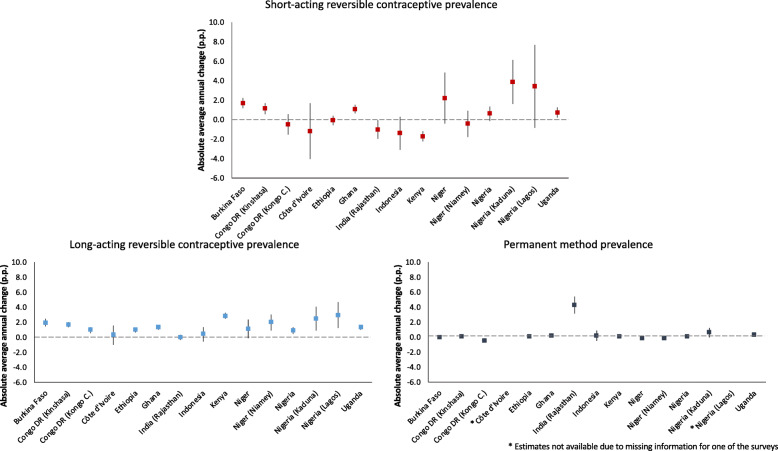
Absolute average annual change of the prevalence of short-acting reversible contraceptives (SARC), long-acting reversible contraceptives (LARC) and permanent methods (PERM) by geography from the first to the most recent PMA2020 survey

Analyzing the trends according to mCPR subtype, it is possible to see that the SARC prevalence increased in five out of the 15 geographies analyzed (Fig. 1). The increases ranged from 0.7 (95% CI: 0.2; 1.3) to 3.9 (95% CI: 1.6; 6.1) percentage points per year in Uganda and Kaduna, Nigeria, respectively. Increases on LARC prevalence were noted in 73% of the geographies analyzed, varying from 0.9 percentage points in Nigeria (95% CI: 0.5; 1.3) to 2.9 percentage points per year in Kenya (95% CI: 2.4; 3.3) and Lagos, Nigeria (95% CI: 1.2; 4.7). Regarding permanent contraception, Rajasthan, India, had a prevalence of sterilization almost 8 times higher than Indonesia, the geography with the lowest prevalence, in 2016 (30.8 and 3.9%, respectively) (Table 2). Still showing a steady progress, our results estimate that the prevalence of permanent methods increased by 4.3 percentage points per year in Rajasthan, India (95% CI: 3.1; 5.4) (Fig. 1). Detailed information about AAAC can be obtained in Additional file 1, while the AAAC of the share of each mCPR subtype is presented in Additional file 3.

**Fig. 2:**
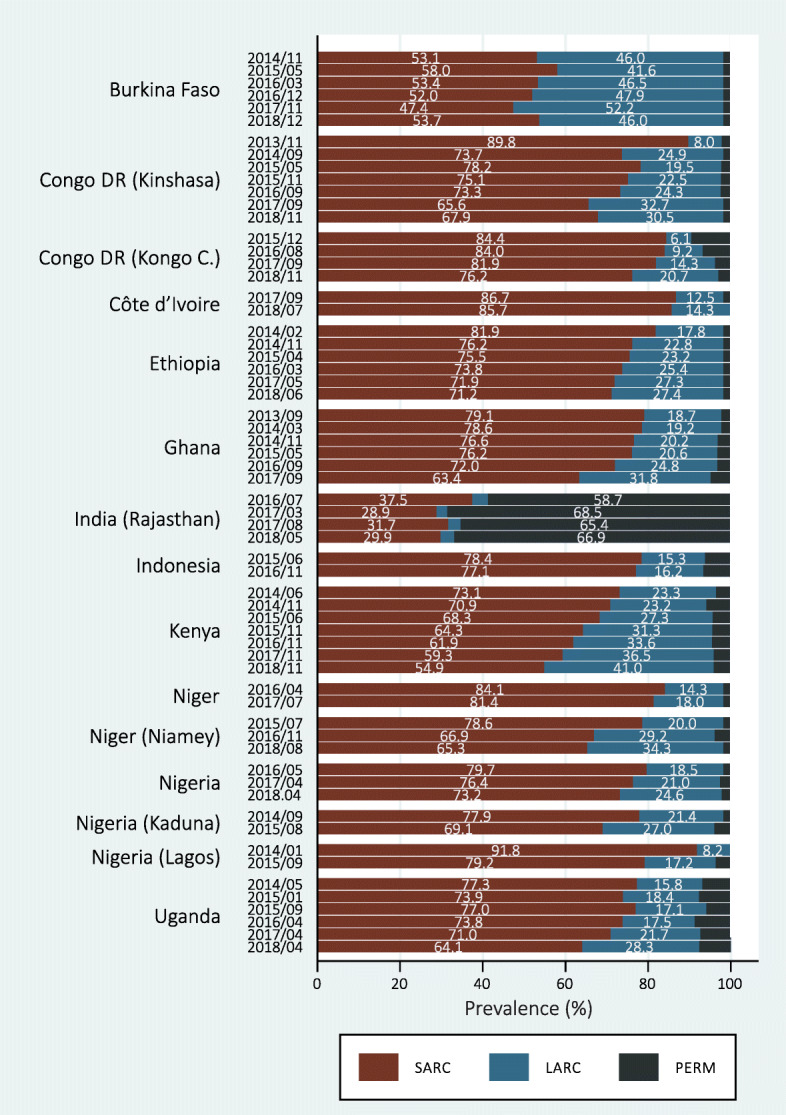
Share of the total prevalence of modern contraceptives use that is represented by short-acting reversible contraception (SARC), long-acting reversible contraception (LARC), and permanent methods (PERM)

Figure 2 shows that in most geographies analyzed, the largest share of mCPR is represented by SARC. But also, in most geographies, the SARC share decreased with time, giving space for LARC. In Kenya, for instance, the SARC share was 73.1% in 2014 and reduced to 54.9% in 2018 (AAAC = − 4.1; 95% CI: − 4.8; − 3.4), while the LARC share was 23.3% in 2014, reaching 41.0% in 2018 (AAAC = 4.3; 95% CI: 3.6; 5.0) (Fig. 2). Generally, as seen in Fig. 2, the lowest share is represented by the permanent methods. The only exception is India, which presents the lowest SARC and LARC shares among all geographies, and most women using modern contraception rely on permanent methods (Fig. 2). The absolute average annual change in the share of contraceptive types used in each country is presented in Additional file 1.

**Fig. 3:**
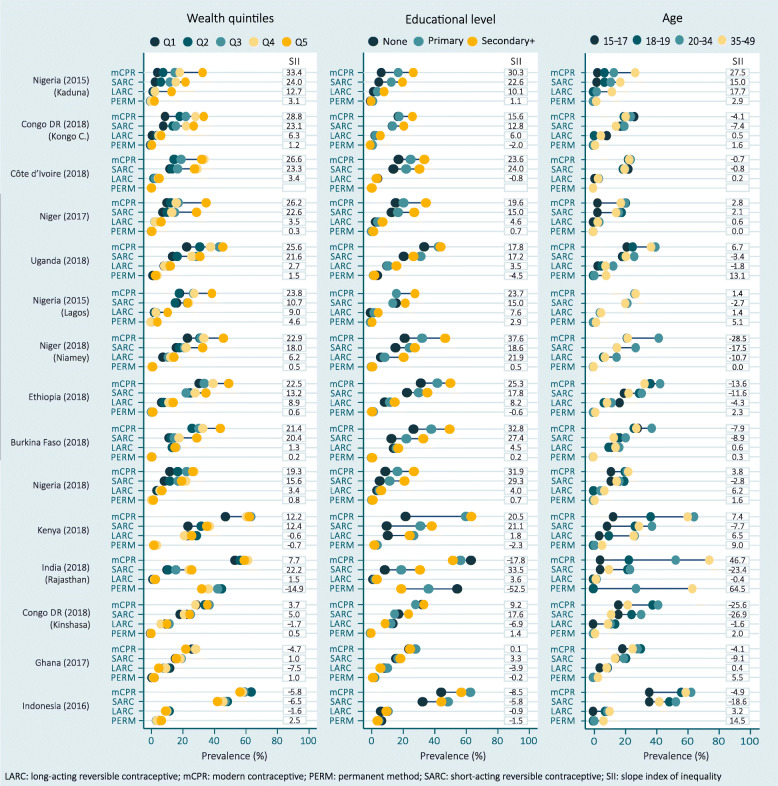
Prevalence of modern contraceptive (mCPR), and its subtypes, by wealth quintiles, educational level and age. Surveys are sorted according to the slope index of inequality (SII) regarding wealth quintiles. Estimates for groups with *N* < 25 are not presented due to their low precision

Absolute wealth inequalities for mCPR were pro-rich in most of the analyzed geographies, which means that the prevalence is higher among wealthier women compared to the poorer ones (Fig. 3 and Additional file 2). There was only one geography where pro-poor inequality was found: Indonesia (SII = − 5.8; 95% CI: − 10.1; − 1.5). The highest inequality was observed in Kaduna, Nigeria, where the prevalence of mCPR among the poorest was 4.3%, while among the richest it reached 32.5% (SII = 33.4; 95% CI: 26.1; 40.7).

Inequalities on the prevalence of mCPR according to educational level ranged from − 17.8 percentage points in Rajasthan, India to 37.6 in Niamey, Niger. In general, the prevalence was higher among women that attained a secondary or higher educational level, and lower among those who did not study (Fig. 3). Only two geographies presented a diverse pattern: India, where the prevalence of mCPR was higher for uneducated women (driven by sterilization), and Indonesia, where the highest prevalence was found among women with primary education.

Considering educational level, inequalities for LARC and permanent methods are generally smaller than for SARC. SARC prevalence is higher among women that attained a higher level of education compared to those who did not study. However, this is not the case for LARC and permanent methods. In Rajasthan, India the prevalence of permanent methods was much higher among women with no education (54.2%) compared to those who reached the secondary level of education or more (18.7%). The corresponding SII − 52.5 percentage points represents the largest absolute inequality observed in the study. It is interesting that, in this case, inequality for education was considerably bigger than wealth inequality.

For most of the surveys analyzed, the older the age group, the higher was the prevalence of mCPR. The highest differences between age groups were observed in Rajasthan, India (SII = 72.0; 95% CI: 61.6; 82.4), with the prevalence of mCPR around 70.0% for the oldest group and around 5.0% for the youngest group (refer to Fig. 3). This geography stands out due to the 61.3% prevalence of permanent methods among women from 35 to 49 years of age, and to the high prevalence (26.5%) of permanent methods among women from 20 to 34 years of age. In the case of permanent methods, these large differences are clearly related to the accumulation of sterilized women along the life cycle, resulting in this large proportion towards the end of the reproductive age. There were less than 25 women with 15 to 17 years of age in Lagos, Nigeria and Niamey, Niger. In Lagos, Nigeria, the group from 18 to 19 years of age also had less than 25 women (refer to Additional file 4). These groups were not considered in the analyses due to their small sample sizes and potential low precision.

According to subtype of mCPR, the highest wealth related SII were found for SARC. Considering all geographies together, the median SII for SARC was 18.0 percentage points, while for LARC and PERM were 3.4 and 0.6 percentage points, respectively. Regarding LARC, the highest inequalities were pro-rich and occurred in Kaduna, Nigeria (SII = 12.7, 95% CI: 5.4; 20.0). For permanent methods, the highest inequalities were pro-poor and seen in India, indicating that sterilization among the poorest women is almost 15 percentage points higher than among the wealthiest ones (SII = − 14.9, 95% CI: − 20.5; − 9.2) (Fig. 3).

## Discussion

Our results showed that the overall mCPR prevalence is increasing in most geographies, but the levels are still low. As mCPR includes all women in its calculation, it is not expected that is will ever reach 100%. We have shown in a previous publication that 80% contraceptive use indicates that nearly all women in need are using contraception [17]. Bearing that in mind, we can say that none of the countries studied are close to the desired universal coverage, since the most recent prevalence observed was always below 60% and, in the worst case, of 22% in Kongo Central, Congo DR. Inequalities persist in favor of richer and better educated women. Even in the groups with the highest prevalence, mCPR is at most 60%, reaching 70% only in a few countries (Kenya, Indonesia and Rajasthan, India). However, in Rajasthan the higher mCPR is due to the high sterilization rates. So, even in the most schooled and richest groups we are still far from the desired goals [8, 9]. SARC were responsible for the largest share of mCPR in most geographies, followed by LARC. However, in general, the share of SARC is decreasing and giving space for LARC, while the share of permanent methods did not vary. The exception was India’s Rajasthan state, where the prevalence of permanent methods was extremely high and increased from 2016 to 2018.

The increase on mCPR is beneficial for maternal and child health by reducing abortions and the risk of morbimortality [2]. Access to family planning strategies can also empower women by increasing their body autonomy and allowing them to decide when and whether to have children, which may facilitate their participation in paid jobs [18]. In the other hand, gender norms might disempower women and prevent them for accessing reproductive health services and contraceptives. For example, expectations that the girls only initiate their sexual life after marriage, which is quite common in many of the countries analyzed, might inhibit unmarried women from looking for these services and use contraception. Increasing access to sexual and reproductive health contributes to control and reduce fertility rates, which in turn can increase the working-age population and positively impact the economic growth of a country [18]. The main reasons for nonuse of contraceptives in LMICs are health concerns and infrequent sex [19]. Lack of access and opposition from the partner are also substantially high in some countries, with the latter being higher among women in a union compared to sexually active not partnered ones. Thus, social norms play an important role in determining mCPR. Another study showed that in many LMICs mCPR is particularly lower among married adolescents with no children compared to unmarried sexually active and those married with children, suggesting that fertility expectations and cultural barriers also have an important role [20]. It is important to act on funding of family planning programs and to make a variety of contraceptive methods accessible to suit each woman needs. It is also essential to increase information, invest on reproductive health education and to work at societal level to change minds, expectations and perspectives.

Increasing financial support to sexual and reproductive health programs is a viable intervention to encourage family planning uptake. Historically, LMICs strongly relied on the support of external funders [21]. However, the recent decrease of funding from donors should be compensated by an increase on government expenditure on sexual and reproductive health to maintain family planning allowance [22]. If the costs are shifted towards users, it is possible that countries experience a plateau or reduction on the prevalence of mCPR. For instance, Congo DR generally requires a fee to be paid by users in order to have access to contraception [23], and the country presented a plateau on mCPR from 2013 to 2017. In a country where 75% of the population lives with less than one dollar per day, the cost of contraception has a major impact on the families’ budget and a low uptake of modern contraception is expected if users have to cover it. Congo DR is still recovering from the consequences of a conflict that ended in 2003, which increased gender inequalities, especially in sexual health and gender-based violence. Many women are not able to discuss family panning with their partner, who usually makes the decisions [24]. Along with allowances to increase modern contraception uptake, providing information and encouraging societal norms which are favorable to family planning can also contribute to a better scenario [23, 25]. However, it is important to think about equitable and sustainable strategies in order to benefit those most in need and avoid increasing the existent inequalities [26].

In all African geographies analyzed, SARC represented the largest share of modern contraception. However, we described a change on this scenario from 2013 to 2018, with a decreasing share of SARC that is giving space for the LARC. The reduction on the prevalence or share of a contraceptive method could be due to a reduction on the number of users, but also because the number of users of a given method did not increase proportionally to the increase on the overall number of modern contraceptive users. Even so, it is important that family planning programs encourage the uptake of LARC, since they are economic and effective alternatives to SARC by not depending on the direct action of the women or their partners.

A very different pattern of modern contraception was observed in Rajasthan, India, where the share of the permanent methods was close to 70% in 2018 (prevalence of 39.1%). Although sterilization might be recommended to some women, these rates are unjustifiably high, given the current availability of low-cost high efficacy methods. India was one of the first countries to create a family planning department, which focused on sterilization [27]. Procedures were conducted on what was called “sterilization camps”, which performed numerous operations per day [27]. Initiatives as the *Mission Parivar Vikas* were launched in 2016 to revert this scenario by providing financial incentives for women to get contraception shots and for health providers to promote it, besides offering free injectables and pills [28]. However, our results showed that the prevalence of permanent methods increased between 2016 and 2018, including among women below 35 years of age (AAAC = 1.5; 95% CI: 0.4; 2.6; data not shown in tables). Also, the SARC prevalence was slightly reduced and the LARC prevalence remained constant. Despite the efforts to provide a wider method mix, the demand for permanent methods is higher than the service availability to perform proper sterilizations in some regions of India [29]. It can be partially explained by the influence of cultural norms and social networks on method choice, especially among less educated and empowered women, who perceive sterilizations as part of their reproductive cycle and are less likely to use contraceptive methods that are different from the most used by their peers. Inequalities on the prevalence of permanent methods in Rajasthan, India were also described, being higher among poorer (SII = − 14.9), older (SII = 64.5) and non-educated women (SII = − 52.5). Considering that, the challenge of reducing the prevalence of permanent methods is even higher, since interventions and educational programs should be designed using an equity lens in order to reach these groups.

We also described inequalities on the prevalence of mCPR, especially for SARC and LARC. In most geographies the prevalence of mCPR, SARC and LARC were in favor of richer, older and better educated women. These findings are in line with other studies [7, 30–33]. The highest wealth inequalities on modern contraception were perceived in Kaduna, Nigeria. Even though the Executive Council of the Federal Government of Nigeria freed contraceptives of all charges to all Nigerian states [34], only small increases were observed in overall mCPR, SARC and LARC prevalence. Some studies described that misinformation and fear of side effects are among the main reasons of modern contraceptives nonuse [30, 35]. This reinforces the idea that interventions focusing only on financial incentives are not enough, being important to also inform women (and their partners) about the importance of modern contraception [32].

Our results also described inequalities by educational level, being the median SII for SARC and LARC equal to 18.2 and 3.6 percentage points, respectively. Studies from Burkina Faso, Kenya and Nigeria described a similar pattern in favor of better educated women [7, 30]. Misinformation, myths and cultural practices that encourage high fertility are among the reasons behind the low prevalence of use of SARC and LARC by non-educated women [30–32].

According to the woman’s age, our results showed that in most geographies the adolescents tend to be left behind in terms of contraception. A previous study has showed that younger adolescents (15–17 years) have lower prevalence of mCPR compared to those aged 18–19 years in Burkina Faso, Nigeria and Ethiopia [7]. A qualitative study in Uganda showed that young people have strongly embedded misconceptions and profound fears which serve as obstacles to initiation and continuation of contraceptive use. Young people placed considerable weight on the side effects of both hormonal and non-hormonal contraceptive methods [31]. It is important to work on education and information on family planning to overthrow myths, and to offer a variety of methods to suit each woman.

Our study is not free of limitations. Information about the use of modern contraceptives was self-reported by women, and this information could be skewed if interviewer bias or social desirability affected the estimates. In order to reduce the probability of bias, PMA2020 surveys rely on female interviewers. However, in some cases the presence of a family member during the interview could still affect responses, especially among young women and those from highly conservative countries.

However, the study also presents some strengths. Firstly, the use of repeated PMA2020 surveys, which ensure the comparability of the estimates throughout the years. Also, the short period of time between each survey allows to visualize updated trends and to monitor the progress of each geography. Secondly, is the evaluation of inequalities according to socio-demographic and economic characteristics of the population. This is important in order to identify which subgroups of women are being left behind, being able to guide future interventions focused on those who are in most need.

## Conclusions

Out of the 15 geographies analyzed, 11 demonstrated an increase in overall modern contraceptive use throughout the years – being the greatest increase in Nigeria. The increase on modern contraceptive use was mainly driven by the uptake of long-acting reversible contraception in replacement of short-acting methods. However, we also found striking inequalities, with the poorer, low educated and the adolescent ones being generally left behind. We are far from reaching the Sustainable Development Goals for universal sexual and reproductive health, which will require a great effort from organizations, policy makers and health providers. It is important that policies are designed focusing on the groups that are being left behind in order to reduce inequalities and achieve universal coverage. More studies are necessary to identify the reasons why some groups of women present much lower contraceptive use, and how much it is related to individual choice, social norms or lack of access, for example. Increasing contraceptive use is a multifaceted issue, being challenging to design effective strategies. Also, issues like gender norms that might disempower women and inhibit them to use contraception will also have to be tackled.

## Supplementary information

**Additional file 1.** Absolute average annual change (AAAC) on prevalence and shares of modern contraceptive subtypes. Table containing detailed information on the absolute average annual change, and 95% confidence intervals, on prevalence and shares of modern contraceptive subtypes.

**Additional file 2.** Detailed information about slope index of inequality (SII) according to subtype of modern contraceptive (mCPR) by wealth quintiles, educational level and age. Table containing detailed information on the slope index of inequality for each subtype of modern contraceptive by wealth quintiles, educational level and age.

**Additional file 3.** Absolute average annual change of the share of short-acting reversible contraceptives (SARC), long-acting reversible contraceptives (LARC) and permanent methods (PERM) by geography from the first to the most recent PMA2020 survey. Description of data: Figure with the absolute average annual change of the share of each subtype of modern contraceptives by geography.

**Additional file 4.** Absolute frequencies of wealth quintiles, educational level, and age for each PMA2020 survey. Table containing absolute and relative frequencies of wealth quintiles, educational level and age. Yellow cells represent subgroups with less than 50 women, while red cells represent subgroups with less than 25 women.

## Data Availability

The data that support the findings of this study are available from the PMA2020 coordinating center but restrictions apply to the availability of these data, which were used under license for the current study, and so are not publicly available. Data are however available from the authors upon reasonable request and with permission of the PMA2020 coordinating center.

## Abbreviations

95% CI: 95% confidence interval
AAAC: Absolute average annual change
FP2020: Family Planning 2020
LARC: Long-acting reversible methods
LMICs: Low- and middle-income countries
mCPR: Modern contraceptive prevalence
PERM: Permanent methods
PMA2020: Performance Monitoring and Accountability 2020
SARC: Short-acting reversible methods
SII: Slope index of inequality
