# Supplementary material for: Socio-demographic and economic inequalities in modern contraception in 11 low- and middle-income countries: an analysis of the PMA2020 surveys
Source: Reprod Health. 2020 Jun 1;17:82. doi: 10.1186/s12978-020-00931-w (PMC7268403; doi:10.1186/s12978-020-00931-w)
Supplement: Supplementary file 1 — Additional file 1. Absolute average annual change (AAAC) on prevalence and shares of modern contraceptive subtypes. Table containing detailed information on the absolute average annual change, and 95% confidence intervals, on prevalence and shares of modern contraceptive subtypes. [file 12978_2020_931_MOESM1_ESM.docx]

Additional file 1. Absolute average annual change (AAAC) on prevalence and shares of modern contraceptive subtypes.

| **Geography** | **Prevalence changes** | | | | | | | | **Share changes** | | | | | |
| --- | --- | --- | --- | --- | --- | --- | --- | --- | --- | --- | --- | --- | --- | --- |
|  | **SARC** | | **LARC** | | | **PERM** | | | **SARC** | | **LARC** | | **PERM** | |
|  | **AAAC** | **95% CI** | | **AAAC** | **95% CI** | | **AAAC** | **95% CI** | **AAAC** | **95% CI** | **AAAC** | **95% CI** | **AAAC** | **95% CI** |
| Burkina Faso | 1.7 | 1.2; 2.2 | | 2.0 | 1.5; 2.5 | | 0.0 | 0.0; 0.0 | -0.9 | -1.3; -0.6 | 1.0 | 0.7; 1.4 | -0.1 | -0.5; 0.2 |
| Congo DR (Kinshasa) | 1.1 | 0.6; 1.7 | | 1.7 | 1.4; 2.0 | | 0.0 | 0.0; 0.1 | -3.2 | -3.7; -2.8 | 3.3 | 2.8; 3.7 | 0.0 | -0.4; 0.4 |
| Congo DR (Kongo C.) | -0.5 | -1.5; 0.6 | | 1.0 | 0.5; 1.4 | | -0.4 | -0.7; -0.1 | -2.7 | -3.5; -1.9 | 5.0 | 4.1; 5.8 | -2.2 | -3.1; -1.4 |
| Côte d'Ivoire | -1.2 | -4.0; 1.7 | | 0.3 | -1.0; 1.5 | | * |  | -1.0 | -3.5; 1.4 | 1.8 | -0.7; 4.2 | -0.7 | -3.2; 1.7 |
| Ethiopia | -0.1 | -0.5; 0.4 | | 1.0 | 0.7; 1.2 | | 0.1 | 0.0; 0.1 | -1.9 | -2.3; -1.6 | 1.8 | 1.5; 2.2 | 0.1 | -0.3; 0.4 |
| Ghana | 1.1 | 0.6; 1.5 | | 1.3 | 1.1; 1.6 | | 0.2 | 0.1; 0.3 | -3.9 | -4.3; -3.6 | 3.3 | 3.0; 3.7 | 0.6 | 0.3; 0.9 |
| India (Rajasthan) | -1.0 | -2.0; -0.1 | | -0.1 | -0.4; 0.3 | | 4.3 | 3.1; 5.4 | -4.0 | -4.8; -3.2 | -0.3 | -1.1; 0.5 | 4.3 | 3.5; 5.1 |
| Indonesia | -1.4 | -3.1; 0.3 | | 0.4 | -0.6; 1.4 | | 0.2 | -0.5; 0.8 | -1.3 | -2.6; 0.1 | 0.9 | -0.5; 2.2 | 0.4 | -1.0; 1.7 |
| Kenya | -1.7 | -2.2; -1.2 | | 2.9 | 2.4; 3.3 | | 0.0 | -0.1; 0.2 | -4.0 | -4.4; -3.7 | 4.2 | 3.8; 4.6 | -0.2 | -0.5; 0.2 |
| Niger | 2.2 | -0.4; 4.9 | | 1.1 | -0.1; 2.3 | | -0.1 | -0.4; 0.2 | -2.7 | -4.8; -0.6 | 3.6 | 1.5; 5.7 | -1.0 | -3.0; 1.1 |
| Niger (Niamey) | -0.4 | -1.8; 0.9 | | 2.0 | 0.9; 3.0 | | -0.2 | -0.3; 0.0 | -3.9 | -4.9; -2.8 | 4.4 | 3.4; 5.5 | -0.6 | -1.6; 0.5 |
| Nigeria | 0.6 | -0.1; 1.3 | | 0.9 | 0.5; 1.3 | | 0.1 | -0.1; 0.2 | -3.3 | -3.8; -2.7 | 3.1 | 2.5; 3.6 | 0.2 | -0.4; 0.7 |
| Nigeria (Kaduna) | 3.9 | 1.6; 6.1 | | 2.4 | 0.8; 4.0 | | 0.6 | -0.1; 1.3 | -8.9 | -10.6; -7.2 | 5.6 | 4.0; 7.3 | 3.2 | 1.6; 4.9 |
| Nigeria (Lagos) | 3.4 | -0.8; 7.7 | | 2.9 | 1.2; 4.7 | | * |  | -12.7 | -16.5; -9.0 | 9.1 | 5.3; 12.8 | 3.7 | -0.1; 7.4 |
| Uganda | 0.7 | 0.2; 1.3 | | 1.3 | 1.0; 1.6 | | 0.3 | 0.1; 0.5 | -3.1 | -3.5; -2.7 | 2.9 | 2.5; 3.3 | 0.2 | -0.2; 0.6 |
| * Estimates not available due to missing information for one of the surveys AAAC: absolute average annual change in percentage points; CI: confidence interval; LARC: long-acting reversible contraceptive; PERM: permanent method; SARC: short-acting reversible contraceptive. | | | | | | | | | | | | | | |
