# Supplementary material for: Socio-demographic and economic inequalities in modern contraception in 11 low- and middle-income countries: an analysis of the PMA2020 surveys
Source: Reprod Health. 2020 Jun 1;17:82. doi: 10.1186/s12978-020-00931-w (PMC7268403; doi:10.1186/s12978-020-00931-w)
Supplement: Supplementary file 2 — Additional file 2. Detailed information about slope index of inequality (SII) according to subtype of modern contraceptive (mCPR) by wealth quintiles, educational level and age. Table containing detailed information on the slope index of inequality for each subtype of modern contraceptive by wealth quintiles, educational level and age. [file 12978_2020_931_MOESM2_ESM.docx]

Additional file 2. Detailed information about slope index of inequality (SII) according to subtype of modern contraceptive (mCPR) by wealth quintiles, educational level and age.

| **Geography** | **Type of contraceptive** | **Wealth quintiles** | | **Educational level** | | **Age** | |
| --- | --- | --- | --- | --- | --- | --- | --- |
|  |  | **SII** | **95% CI** | **SII** | **95% CI** | **SII** | **95% CI** |
| Nigeria (2015) (Kaduna) | mCPR | 33.4 | 26.1; 40.7 | 30.3 | 22.6; 38.0 | 27.5 | 19.0; 36.1 |
|  | SARC | 24.0 | 17.4; 30.5 | 22.6 | 15.4; 29.8 | 15.0 | 7.3; 22.7 |
|  | LARC | 12.7 | 5.4; 20.0 | 10.1 | 4.1; 16.0 | 17.7 | 9.6; 25.8 |
|  | PERM | 3.1 | -0.3; 6.5 | 1.1 | 0.0; 2.2 | 2.9 | -0.7; 6.5 |
| Congo DR (2018) (Kongo C.) | mCPR | 28.8 | 20.6; 37.0 | 15.6 | 5.5; 25.6 | -4.1 | -13.8; 5.5 |
|  | SARC | 23.1 | 15.1; 31.0 | 12.8 | 3.5; 22.2 | -7.4 | -16.1; 1.3 |
|  | LARC | 6.3 | 2.2; 10.5 | 6.0 | 0.4; 11.5 | 0.5 | -4.3; 5.3 |
|  | PERM | 1.2 | -0.4; 2.9 | -2.0 | -3.7; -0.3 | 1.6 | -0.3; 3.5 |
| Côte d'Ivoire (2018) | mCPR | 26.6 | 19.7; 33.5 | 23.6 | 16.4; 30.9 | -0.7 | -8.5; 7.0 |
|  | SARC | 23.3 | 16.6; 30.0 | 24.0 | 17.1; 30.9 | -0.8 | -8.3; 6.7 |
|  | LARC | 3.4 | 0.4; 6.4 | -0.8 | -3.8; 2.2 | 0.2 | -2.6; 3.0 |
|  | PERM | 0.0 | 0.0; 0.0 | 0.0 | 0.0; 0.0 | 0.0 | 0.0; 0.0 |
| Niger (2017) | mCPR | 26.2 | 19.4; 33.0 | 19.6 | 12.2; 26.9 | 2.8 | -4.6; 10.1 |
|  | SARC | 22.6 | 16.3; 29.0 | 15.0 | 8.0; 21.9 | 2.1 | -4.7; 9.0 |
|  | LARC | 3.5 | 0.5; 6.6 | 4.6 | 1.5; 7.7 | 0.6 | -2.4; 3.5 |
|  | PERM | 0.3 | 0.0; 0.7 | 0.7 | -0.3; 1.8 | 0.0 | -0.2; 0.3 |
| Uganda (2018) | mCPR | 25.6 | 18.8; 32.4 | 17.8 | 9.0; 26.6 | 6.7 | -0.9; 14.4 |
|  | SARC | 21.6 | 15.4; 27.8 | 17.2 | 9.5; 24.9 | -3.4 | -10.2; 3.4 |
|  | LARC | 2.7 | -1.9; 7.3 | 3.5 | -2.3; 9.3 | -1.8 | -6.0; 2.4 |
|  | PERM | 1.5 | -0.7; 3.7 | -4.5 | -8.1; -0.9 | 13.1 | 8.9; 17.3 |
| Nigeria (2015) (Lagos) | mCPR | 23.8 | 13.6; 34.0 | 23.7 | 9.1; 38.3 | 1.4^¥^ | -10.3; 13.2 |
|  | SARC | 10.7 | 1.3; 20.2 | 15.0 | 1.2; 28.7 | -2.7^¥^ | -13.6; 8.1 |
|  | LARC | 9.0 | 2.9; 15.1 | 7.6 | 0.1; 15.2 | 1.4^¥^ | -4.0; 6.9 |
|  | PERM | 4.6 | 0.6; 8.5 | 2.9 | 0.3; 5.5 | 5.1^¥^ | -2.2; 12.4 |
| Niger (2018) (Niamey) | mCPR | 22.9 | 11.2; 34.5 | 37.6 | 25.7; 49.5 | -28.5^¥^ | -43.2; -13.7 |
|  | SARC | 18.0 | 8.0; 28.0 | 18.6 | 8.0; 29.2 | -17.5^¥^ | -30.2; -4.9 |
|  | LARC | 6.2 | -2.8; 15.1 | 21.9 | 10.9; 32.9 | -10.7^¥^ | -21.6; 0.2 |
|  | PERM | 0.5 | -0.5; 1.6 | 0.5 | -0.5; 1.4 | 0.0^¥^ | 0.0; 0.0 |
| Ethiopia (2018) | mCPR | 22.5 | 16.9; 28.1 | 25.3 | 19.5; 31.2 | -13.6 | -20.4; -6.8 |
|  | SARC | 13.2 | 7.8; 18.6 | 17.8 | 12.3; 23.3 | -11.6 | -17.9; -5.4 |
|  | LARC | 8.9 | 5.5; 12.4 | 8.2 | 4.5; 12.0 | -4.3 | -8.5; 0.0 |
|  | PERM | 0.6 | -0.5; 1.6 | -0.6 | -1.6; 0.4 | 2.3 | 0.8; 3.8 |
| Burkina Faso (2018) | mCPR | 21.4 | 14.9; 27.9 | 32.8 | 25.8; 39.7 | -7.9 | -15.4; -0.4 |
|  | SARC | 20.4 | 15.0; 25.8 | 27.4 | 21.7; 33.2 | -8.9 | -14.8; -3.0 |
|  | LARC | 1.3 | -3.8; 6.4 | 4.5 | -1.5; 10.5 | 0.6 | -5.0; 6.2 |
|  | PERM | 0.2 | -0.1; 0.5 | 0.2 | -0.3; 0.6 | 0.3 | -0.2; 0.9 |
| Nigeria (2018) | mCPR | 19.3 | 15.4; 23.1 | 31.9 | 27.2; 36.5 | 3.8 | -0.5; 8.2 |
|  | SARC | 15.6 | 12.2; 19.1 | 29.3 | 24.8; 33.8 | -2.8 | -6.7; 1.1 |
|  | LARC | 3.4 | 1.2; 5.6 | 4.0 | 1.4; 6.6 | 6.2 | 3.8; 8.6 |
|  | PERM | 0.8 | -0.2; 1.7 | 0.7 | -0.2; 1.5 | 1.6 | 0.6; 2.6 |
| Kenya (2018) | mCPR | 12.2 | 6.4; 18.0 | 20.5 | 14.2; 26.8 | 7.4 | 0.4; 14.4 |
|  | SARC | 12.4 | 6.9; 17.9 | 21.1 | 15.2; 27.1 | -7.7 | -14.1; -1.3 |
|  | LARC | -0.6 | -5.9; 4.7 | 1.8 | -3.9; 7.6 | 6.5 | 0.6; 12.3 |
|  | PERM | -0.7 | -2.4; 0.9 | -2.3 | -4.6; -0.1 | 9.0 | 5.9; 12.0 |
| India (2018) (Rajasthan) | mCPR | 7.7 | 1.9; 13.5 | -17.8 | -23.7; -11.9 | 46.7 | 41.4; 52.0 |
|  | SARC | 22.2 | 17.7; 26.7 | 33.5 | 28.8; 38.1 | -23.4 | -28.5; -18.3 |
|  | LARC | 1.5 | -0.1; 3.1 | 3.6 | 1.7; 5.6 | -0.4 | -2.2; 1.4 |
|  | PERM | -14.9 | -20.5; -9.2 | -52.5 | -57.1; -47.9 | 64.5 | 60.5; 68.5 |
| Congo DR (2018) (Kinshasa) | mCPR | 3.7 | -5.7; 13.0 | 9.2 | -6.6; 25.0 | -25.6 | -36.1; -15.0 |
|  | SARC | 5.0 | -3.1; 13.1 | 17.6 | 2.6; 32.5 | -26.9 | -35.9; -17.9 |
|  | LARC | -1.7 | -8.0; 4.7 | -6.9 | -16.1; 2.2 | -1.6 | -9.4; 6.2 |
|  | PERM | 0.5 | -0.8; 1.9 | 1.4 | 0.2; 2.6 | 2.0 | 0.3; 3.7 |
| Ghana (2017) | mCPR | -4.7 | -10.9; 1.6 | 0.1 | -6.6; 6.8 | -4.1 | -10.9; 2.6 |
|  | SARC | 1.0 | -4.2; 6.1 | 3.3 | -2.3; 9.0 | -9.1 | -14.8; -3.5 |
|  | LARC | -7.5 | -11.3; -3.7 | -3.9 | -7.8; 0.0 | 0.4 | -3.8; 4.6 |
|  | PERM | 1.0 | -0.6; 2.5 | -0.2 | -1.3; 1.0 | 5.5 | 3.1; 7.9 |
| Indonesia (2016) | mCPR | -5.8 | -10.1; -1.5 | -8.5 | -13.4; -3.5 | -4.9 | -9.8; 0.0 |
|  | SARC | -6.5 | -10.9; -2.2 | -5.8 | -10.9; -0.8 | -18.6 | -23.4; -13.8 |
|  | LARC | -1.6 | -4.1; 1.0 | -0.9 | -3.9; 2.1 | 3.2 | 0.3; 6.2 |
|  | PERM | 2.5 | 0.6; 4.4 | -1.5 | -3.5; 0.6 | 14.5 | 11.4; 17.5 |
| ^¥^ Some groups were not considered in the analysis due to the low precision of estimates (N<25)  mCPR: modern contraceptive; PERM: permanent method; LARC: long-acting reversible contraceptive; SARC: short-acting reversible contraceptive; SII: slope index of inequality | | | | | | | |
