# Supplementary figures and images for: Socio-demographic and economic inequalities in modern contraception in 11 low- and middle-income countries: an analysis of the PMA2020 surveys
Source: Reprod Health. 2020 Jun 1;17:82. doi: 10.1186/s12978-020-00931-w (PMC7268403; doi:10.1186/s12978-020-00931-w)

## Slide 1
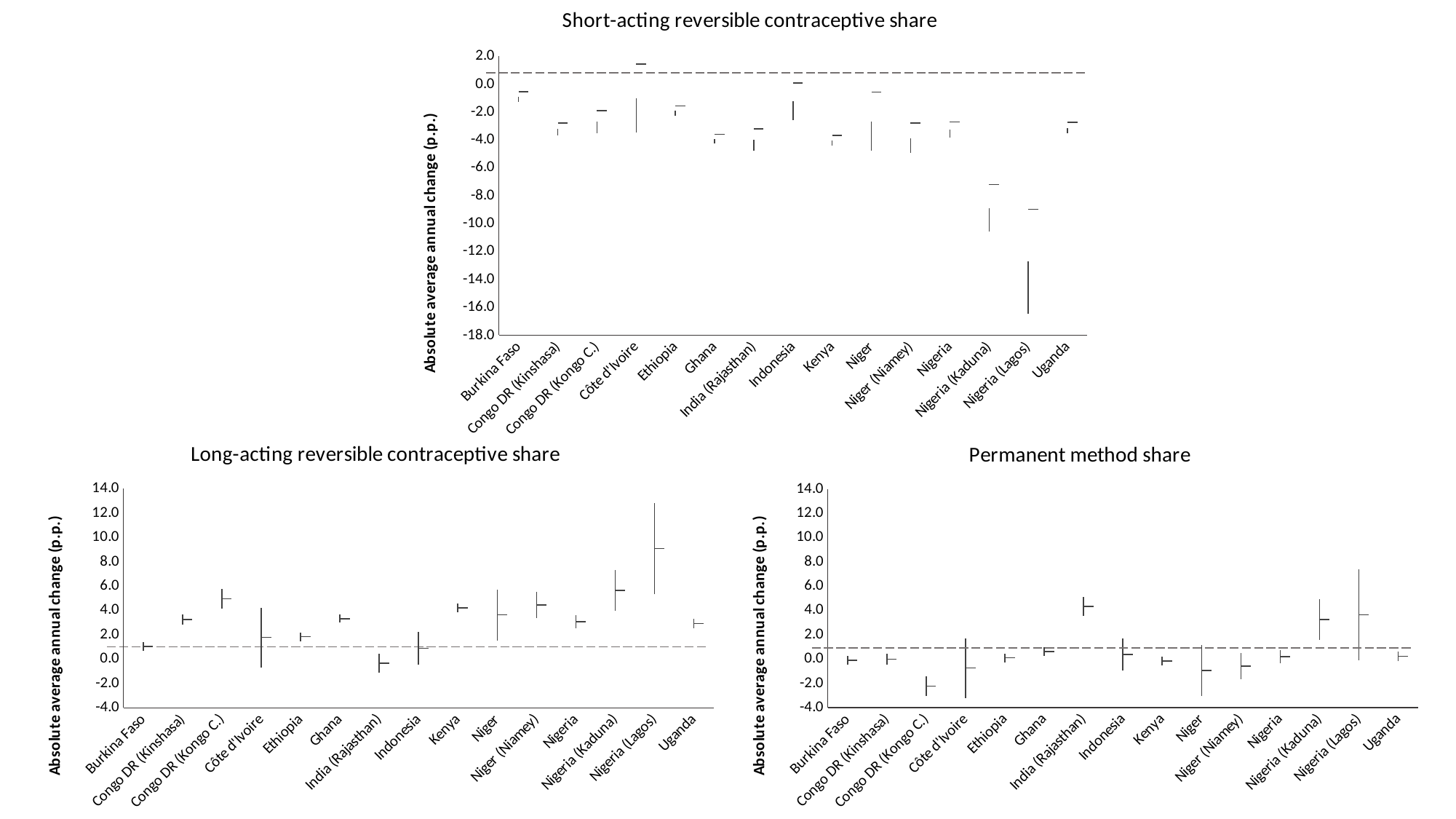

[unsupported chart]
[unsupported chart]
[unsupported chart]

Supplement: Supplementary file 3 — Additional file 3. Absolute average annual change of the share of short-acting reversible contraceptives (SARC), long-acting reversible contraceptives (LARC) and permanent methods (PERM) by geography from the first to the most recent PMA2020 survey. Description of data: Figure with the absolute average annual change of the share of each subtype of modern contraceptives by geography. [file 12978_2020_931_MOESM3_ESM.pptx]
